# Scabies vaccines: where we stand and challenges ahead

**DOI:** 10.1007/s00436-024-08298-8

**Published:** 2024-07-24

**Authors:** Mahmoud S. Sharaf

**Affiliations:** Parasitology Department, Faculty of Medicine, Tanta University, Tanta, Gharbia Egypt

**Keywords:** Scabies, *Sarcoptes scabiei*, Immunity, Treatment, Vaccines

## Abstract

Scabies is an itchy skin disease caused by the burrowing mite, *Sarcoptes scabiei*. During their lifespan, female mites invade the stratum corneum and create tunnels in which they reside, move, feed, deposit fecal pellets, and lay eggs. Globally, more than 200 million people are estimated to be affected by scabies annually. Currently, using scabicidal agents is the only approved method for treating scabies. However, resistance to commonly used agents such as permethrin and ivermectin has been observed in scabies mites. Therefore, the development of vaccines for scabies, either as a preventative measure or for treatment, is crucial to control such neglected diseases. Since the host could evolve a protective immune response that could prevent re-infestation by scabies mites, vaccine development is theoretically possible. This review aims to provide a comprehensive overview of the ongoing challenges regarding the currently available control measures for scabies. It also explores the promising path of scabies vaccine development, highlighting the current state of research and challenges that need to be addressed to develop new and innovative measures for both treating and preventing scabies infections.

## Background

Scabies is an itchy skin disease caused by a burrowing mite called *Sarcoptes scabiei* (*S. scabiei*). It is now included in the World Health Organization roadmap for neglected tropical diseases 2021–2030. Globally, it is estimated to affect more than 200 million individuals at any given time and more than 400 million individuals cumulatively every year (WHO 2024). While scabies can affect individuals of any age, it is particularly prevalent among children and the elderly residing in low-resource areas. Although scabies is more common in tropical countries, it can also occur in other regions with cold weather due to increased direct personal contact and increased mites’ survival (Liu et al. 2016). Scabies is more likely to spread in crowded environments and is not necessarily a result of poor hygiene. This is why it is common in places like prisons, military camps, and boarding schools (Del Campillo et al. 2021).

**Fig. 1:**
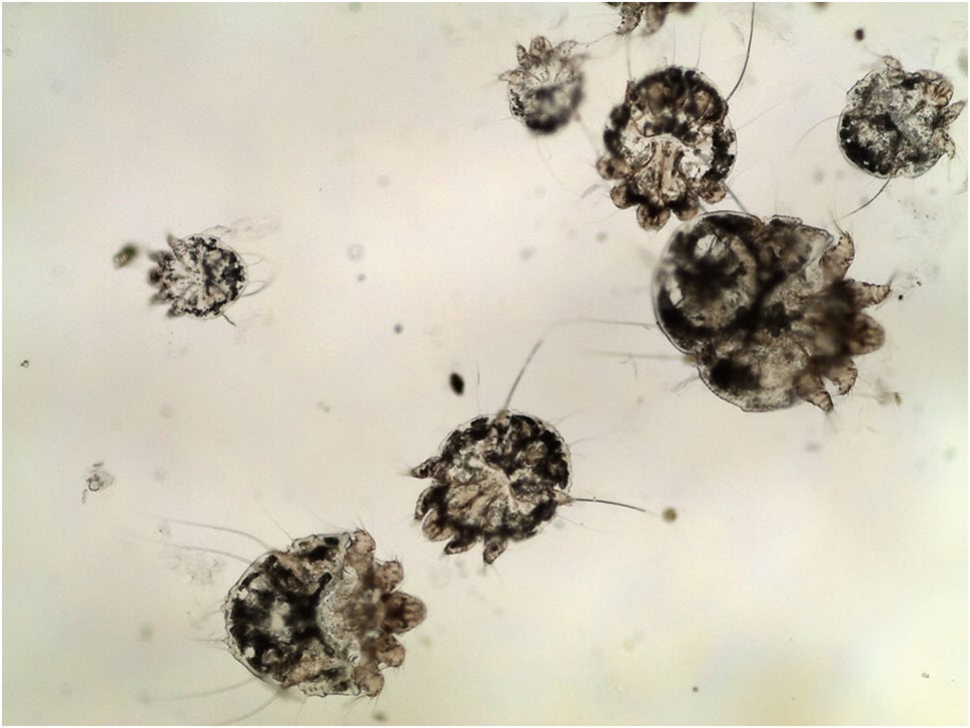
Microscopic appearance motile stages of scabies mites (× 100)

*Sarcoptes scabiei* goes through five developmental stages in its life cycle, including egg, larva, protonymph, tritonymph, and adult. The adult scabies mite has a characteristic tortoise-like body, with a gnathosoma anteriorly and an idiosoma posteriorly. The gnathosoma is the head-like structure that surrounds the mouth opening, while the idiosoma is the oval body of the mite, which is ventrally flat and dorsally arched, with four pairs of legs arising from its ventral aspect. The dorsal surface of the idiosoma is not smooth, since it shows transversely arranged ridges, scales, setae, and spines (Sharaf et al 2023b) (Fig. 1).

During its lifespan (4–6 weeks), the fertilized female lays eggs within the burrow formed in the stratum corneum. After hatching, the released larvae dig new pocket burrows and wander on the surface of the host’s body until maturation. Secondary bacterial infections can lead to serious health issues like sepsis, glomerulonephritis, and rheumatic fever. As a result, many countries have considered scabies as a public health concern that requires effective intervention (van der Linden et al. 2019).

## The entangled dance between immunity and the scabies mite

The immune response to scabies mites involves coordinated responses between different immune system components like natural killer cells, macrophages, neutrophils, dendritic cells, mast cells, eosinophils, lymphocytes, antibodies, complement system, and different cytokines. Although the exact roles and ideal balance of these components for complete protection are not fully understood, it is now well established that the clinical severity of the disease depends on the type and the amplitude of the associated cellular and humoral immune responses (Mounsey et al. 2015; Abd El-Aal et al. 2016; Sharaf et al. 2023b).

Generally, there are two main forms of scabies: ordinary scabies (OS) and crusted scabies (CS). Ordinary scabies is characterized by a protective local immune response dominated by CD4^+^ T-lymphocytes with a T helper-1 (Th1) cytokine profile, including interferon-gamma (IFN-γ), tumor necrosis factor-alpha (TNF-α), and interleukin-2 (IL-2), while the immune response in CS is non-protective and dominated by CD8^+^ T-lymphocytes and a Th2 cytokine profile, including IL-4, IL-5, and IL-13. Th17 cells are a crucial subset of T-cells that contribute to the local immune response in CS by recruiting and activating neutrophils at sites of inflammation, as well as stimulating endothelial cells, keratinocytes, and fibroblasts to produce inflammatory cytokines like IL-1, IL-6, and TNF-α. Their role in CS is supported by high levels of IL-17 and IL-23 (Mounsey et al. 2015).

T-regulatory lymphocytes (Treg) could potentially contribute to the immune response against *Sarcoptes scabies* mites by suppressing the inflammatory reaction to the mite through the secretion of IL-10 and the inhibition of proinflammatory cytokines, such as TNF-α, IFN-γ, and IL-2 (Liu et al. 2014). This could, in part, explain the incubation period of a primary infestation caused by *S. scabiei.* Moreover, there is a negative correlation between the activity of Treg cells and the severity of lesions in scabies (Abd El-Aal et al. 2016). This is why skin pathology is severe in cases of CS, where IL-10 production is significantly reduced, leading to the expansion of Th17 cells and a dysfunctional immune response involving Treg/Th17 cells (Gonzalez-Lombana et al. 2013).

*Sarcoptes scabiei* mites have been reported to trigger strong antibody-mediated immune responses, especially in cases with crusted forms of scabies, which have extremely high levels of antigen-specific IgG and IgE when compared with the ordinary form. Such difference can be attributed to the high levels of IL-4 and IL-13 in CS, which play crucial roles in B-cell class switching and the induction of IgE and IgG4 co-expression. However, the timing of the antibody-mediated immune responses and their relative importance in establishing protective immunity are still poorly defined (Walton et al. 2010; Arlian et al. 2015).

Scabies mites have developed various evasion strategies to evade the host’s immune responses, making it difficult for the host to fight the mite. Live scabies mites induce skin fibroblasts and keratinocytes, which are the major cell types in the skin, to release the anti-inflammatory cytokine interleukin-1 receptor antagonist (IL-1ra). This cytokine binds to the IL-1 receptor present in a variety of cells, including T-cells, B-cells, natural killer cells, macrophages, and neutrophils, thereby inhibiting the activity of the proinflammatory cytokine IL-1. This makes it easier for the mites to survive on the host’s skin without being damaged by immune mechanisms (Morgan et al. 2013).

It has also been reported that scabies mite extract stimulates human T-regulatory cells to produce IL-10, a potent anti-inflammatory cytokine. IL-10 reduces the release of proinflammatory cytokines and the expression of MHC-II molecules on antigen-presenting cells, which in turn inhibits or reduces the interaction between the MHC-II-antigen complex and the T-cell receptor necessary for B-cell activation and differentiation into antibody-secreting plasma cells (Arlian et al. 2006). Furthermore, exposure to live scabies mites and their extracts has been reported to increase the production of vascular endothelial growth factor in human skin equivalents and monocultures of normal human epidermal keratinocytes and dermal fibroblasts. This growth factor would likely promote vascularity and the influx of fluid (plasma) to the area around the mite’s mouthparts within the burrow, providing the mite with essential water and nutrients from the otherwise arid stratum corneum (Morgan and Arlian 2010).

*Sarcoptes scabiei* are known to utilize gut serine protease inhibitors to attach to numerous plasma proteins that are part of the complement activation pathways, thereby blocking the classical, alternative, and lectin pathways of the human complement system. As mites feed on plasma, deactivating the host’s complement protects their gut from damage mediated by the complement system. Overall, the intricate interactions between mites and host immune mechanisms highlight the complex arms race between parasites and their hosts. Understanding these mechanisms is crucial for developing effective strategies to combat mite infestations and related skin conditions (Fernando et al. 2023).

## Time to rethink scabies control: why vaccines should be our top priority?

### Challenges regarding diagnosis

Clinical signs and microscopic evaluation of skin scrapings are currently used for the diagnosis of scabies, although experience has shown that these traditional methods have a sensitivity of less than 50%. It might be challenging to detect visible lesions since they are often masked by eczema or impetigo or are atypical. Additionally, the disease can exhibit different clinical forms, including classic scabies, crusted scabies, nodular scabies, bullous scabies, scabies incognito, and animal scabies. These forms can be easily confused with other skin conditions such as eczema, allergic dermatitis, or even insect bites (Thompson et al. 2017).

A typical history of contact with other scabies patients, pruritus that worsens at night, and the distribution of inflammatory papules can all be used to make a presumptive diagnosis. Presumptive therapy is sometimes used to diagnose scabies, but its reliability is uncertain since a favorable response to treatment does not definitively confirm scabies, as other skin conditions can also improve spontaneously. Conversely, a lack of response to treatment does not necessarily rule out scabies, especially in cases of treatment-resistant mites (Engelman et al. 2018).

Although a definitive diagnosis of scabies can be obtained by identifying mites, eggs, eggshell pieces, or mite fecal pellets in scrapings obtained from skin lesions, the sensitivity of this approach for diagnosing classic scabies is low owing to the limited number of mites in such cases. The accuracy of the diagnosis can also be influenced by factors such as the clinical appearance of the skin lesions (unscratched lesions are more useful), the number of sites sampled, and the expertise of the person collecting the samples. A negative test result does not necessarily rule out scabies, especially if the scraping is not done from an active burrow (Kandi 2017; Thompson et al. 2017).

A skin biopsy can also confirm the diagnosis of scabies if the mites or portions of it are found during histopathological evaluation. However, detecting mites can be difficult, and even an expert’s negative test does not rule out scabies since selecting the right site for sampling is critical. Skin biopsy is an invasive procedure and time-consuming (may take 2–7 days to obtain a result). In many cases, the histological appearance is nonspecific, usually showing delayed hypersensitivity with superficial and deep perivascular inflammatory eosinophilic and mononuclear cell infiltrates, papillary edema, and epidermal spongiosis. Hence, it is not used as a routine test for diagnosing scabies and is reserved for the confirmation of challenging or atypical cases (Foo et al. 2013).

Although dermoscopy can be helpful in diagnosing scabies, there are some limitations that hamper its use, particularly for inexperienced operators. One drawback is that the low magnification can make it hard to differentiate between dermoscopic signs of scabies and artefacts caused by scratching, crusts, bleeding, or microscopic dirt particles. Additionally, the low magnification does not allow the detection of eggs and feces, which are sometimes the only diagnostic signs. Furthermore, the dermoscopic signs of scabies are hardly visible on dark skin, limiting the effectiveness of dermoscopy in many countries, and in hairy body areas, where a clear visualization of the skin may be restricted. Finally, using handheld dermoscopy in sensitive areas like the genital region may cause discomfort for both the patient and the healthcare provider (Dupuy et al. 2007; Micali and Lacarrubba 2011; Walter et al. 2011).

Developing a diagnostic serological blood test for scabies is complicated by the problem of co-sensitization or cross-reactivity with antigens from ubiquitous house dust mites, such as *Dermatophagoides farinae*, *Dermatophagoides pteronyssinus*, and *Euroglyphus maynei*. House dust mites have been reported to produce over 25 antigenic proteins, many of which cross-react with those from scabies mites, as reported by Chan et al. (2015). In a study conducted by Arlian et al. (2015) to profile the *Sarcoptes* mite-specific antibodies present in the sera of 91 patients with ordinary scabies, all patients except one showed circulating Ig, IgG, and/or IgM to all three house dust mite extracts, and no scabies patient had antibodies exclusively to scabies mites. Since a significant percentage of people are usually sensitized to the ubiquitous house dust mites, therefore, identifying a limited and defined set (used as a cocktail) of scabies proteins (or protein fragments) that do not share cross-reacting epitopes with those on house dust mite proteins is crucial for developing a diagnostic test that possesses both specificity and sensitivity. To achieve this goal, a detailed and comprehensive proteomic and genomic analysis of scabies mites is necessary.

Other newer diagnostic tests like polymerase chain reaction or isothermal amplification techniques are being explored; however, such tests are not yet widely available or definitive (Fraser et al. 2018; Hahm et al. 2018). Until now, scabies diagnosis largely depends on a high index of suspicion, careful examination, utilizing various diagnostic tools, and consulting with a dermatologist experienced in diagnosing scabies.

### Challenges regarding treatment

The treatment of scabies should include the pharmacological elimination of mites, preventing the infestation from spreading to contacts and managing any complications that may arise (e.g., secondary bacterial infections). Theoretically, *Sarcoptes scabiei* mites can be effectively targeted with a single therapy since they do not have free-living stages or intermediate hosts in their life cycle. However, most scabicides currently available do not kill the eggs, allowing the life cycle to continue and potentially worsen the infection. Additionally, the epidermal cell growth shifts eggs away from the dermal host defense system and/or the systemic acaricidal agents diffusing from the dermis, making it difficult to kill the mites (Sharaf et al. 2023b).

Unless the given non-ovicidal scabicide has a long half-life that spans the mite’s whole cycle, or multiple doses are given at the appropriate times, eggs and newly hatched larvae will not be affected, allowing the infection to progress. Consequently, new scabicides are urgently needed, particularly those that could kill all the developmental stages of the scabies mite. The availability of such medications would improve control strategies and decrease scabies recurrence rates (Bernigaud et al. 2016; Sharaf et al. 2020).

Currently, there are just a few treatment options for human scabies. Traditional broad-spectrum drugs of choice, like permethrin and ivermectin (IVR), are still used mostly owing to their low cost. Currently, ivermectin is the only oral scabicide available for human use, mostly used to treat crusted scabies. Unfortunately, ivermectin has a short half-life in the skin, and multiple doses are generally needed for the effective management of scabies (Bernigaud et al. 2016). Additionally, it is not approved for infants and children less than 15 kg. Furthermore, its safety for pregnant women is still uncertain, so it is recommended to be avoided during pregnancy. Instead, topical scabicidal agents should be considered the preferred option in such cases (Nicolas et al. 2020). Another ongoing issue with IVR is the evidence of treatment failure despite multiple doses, as well as in vitro resistance. As a result, IVR monotherapy is not recommended for cases of crusted scabies. It is recommended to use a combination of IVR with a topical acaricide like permethrin and keratolytic supplements in such cases (Niode et al. 2022).

Although various topical acaricidal agents are available for treating human scabies (e.g., permethrin 5% cream, benzyl benzoate 10–25% lotion or emulsion, crotamiton 10% cream, and precipitated sulfur cream or lotion), they must be applied to the entire body, with special attention to certain areas like the scalp, groin, interdigital spaces, nails, and behind the ears. Furthermore, repeated applications are generally indicated for effective management of scabies. Such a method is exhausting, time-consuming, and not preferable by most patients. Consequently, it is not ideal especially for mass drug administration during outbreaks of scabies (Salavastru et al. 2017). Additionally, resistance to various topical scabicides, including permethrin, lindane, and crotamiton, has been reported (Bernigaud et al. 2020). The use of molecular tools could help identify drug resistance early and accurately, as well as detect genetic changes associated with resistance.

In contrast to the human scenario, where acaricides are few, more acaricidal agents are available for the treatment of sarcoptic mange in livestock and companion animals. Furthermore, the vast majority of medications utilized in humans are taken from the veterinary arsenal. The clear disparity between human and animal scabies treatment availability is mostly due to the companion animal and livestock health sectors being a more profitable market for companies than the neglected tropical diseases sector. Recently, treatment of sarcoptic mange in domestic animals (particularly cats and dogs) has moved to moxidectin and the novel family of isoxazolines, a novel class of acaricides that includes afoxolaner, fluralaner, sarolaner, and lotilaner, due to their documented safety, efficacy, and long-lasting protection of the animal. Hence, such agents may be a promising target for human scabies research (Sharaf et al. 2020, 2023a, 2023b).

## The scabies vaccine enigma: will we finally kick the itch?

As mentioned before, using scabicidal agents is the only approved method for treating scabies until now, and resistance against commonly used agents such as permethrin and ivermectin has been observed in scabies mites. Therefore, the development of vaccines for scabies, either as a preventative measure or for treatment, is crucial to control such neglected diseases. Since the host could evolve a protective immune response against the scabies mites, vaccine development is theoretically possible (Abd El-Aal et al. 2016). Unfortunately, there are no available approved effective scabies vaccines until now owing to the complexity of the host-parasite interactions in scabies, lack of adequate knowledge about the protective immune mechanisms employed by the host, and the vast array of proteins encoded by the parasite, which makes it challenging to identify proteins that could induce protective immunity (Shen et al. 2023). Designing and developing a successful vaccine against *Sarcoptes scabiei* mites is currently a subject of extensive research.

Various studies have been conducted to develop vaccines against scabies mites, including whole-body extract vaccines (Tarigan and Huntley 2005), DNA vaccines (Gu et al. 2014), and recombinant protein vaccines. For a complex parasite like *Sarcoptes scabiei*, immunization with a total extract of scabies mites would be advantageous over recombinant proteins since all structural and immunogenic characteristics that are native to the mite are displayed in the vaccine. However, the in vitro cultivation of the mite is not feasible until now, making the availability of native proteins at the required quantity challenging. Hence, recombinant protein subunit vaccines are easily available candidates to test the protective potential of various immunodominant antigens of the mite (Casais et al. 2016).

Chitinase-like proteins (CLPs) and serine protease inhibitors (serpins) are reported to be involved in various fundamental physiological functions in mites and are also involved in the host-parasite interaction (Sutherland et al. 2009; Mika et al. 2012; Swe and Fischer 2014). In a recent study on mangy rabbits, Shen et al. (2018) reported that immunization with recombinant *S. scabiei* chitinase-like protein 5 (rSs-CLP5) induced a protective immune response, evidenced by high levels of specific antibodies and reduced mite burden. In another study on mangy rabbits, Shen et al. (2020) used a mixture of rSs-CLP5 and rSs-CLP12 for immunization. Interestingly, the authors reported that the rabbit’s immune protection rate and the rate of mite reduction improved, and concluded that a combination of different candidate vaccine molecules could enhance the level of immune protection provided by the vaccine. Along similar lines, Shen et al. (2023) used a cocktail of three recombinant vaccine candidate molecules, rSsCLP5, rSsCLP12, and rSs-serpin, to immunize mangy rabbits, and reported that the protection rate was higher than the effects of combined use of rSsCLP5 and rSsCLP12.

Surprisingly, Casais et al. (2016) reported that no significant protective response was induced when a mixture of two recombinant antigens, Ssλ15 and Ssλ20∆B3, was used to immunize mangy rabbits although high levels of antibodies were noticed after vaccination. Instead, immunized rabbits exhibited higher lesion scores, indicating that the vaccine-induced exacerbated immune response shared in the pathogenesis of such lesions. The lack of protection from vaccination may be related to the type and the levels of the specific antibodies produced by the candidate recombinant protein. Different recombinant vaccines can induce varying levels of protective antibodies, with some being effective in providing immune protection while others are not. Shen et al. (2023) suggested that protective response is possibly related to the levels of the specific IgE antibodies produced after immunization. Furthermore, coordination of IgG and IgE is essential for such a protective response to be accomplished. Hence, hosts immunized with specific *S. scabiei* recombinant protein fractions that induce high levels of specific IgG antibodies but fail to induce specific IgE usually fail to elicit a protective response against scabies mites. Likewise, vaccination with a mixture of rSsCLP5 and rSsCLP12 induced high levels of IgG and IgE antibodies in rabbits, with subsequent protective response against the scabies mites (Shen et al. 2020).

Multi-epitope peptide vaccines are another attractive option for inducing a specific immune response, being cost effective and easy to synthesize and use, with minimal risk of antigen-induced anaphylaxis (Li et al. 2014). Since peptides alone are not very effective at stimulating an immune response, adjuvants are often added to enhance their immunogenicity (Mittal et al. 2020). The GPGPG linkers are commonly used since they aid in presenting epitopes to the immune system and enhance effective immune processing (Saadi et al. 2017). Additionally, they keep the epitopes separate and do not allow them to fold (Nezafat et al. 2014).

Paramyosin, a structural protein only found in invertebrate muscles and involved in immune regulation, is another promising vaccine candidate. Using immunoinformatics tools, Naz et al. (2020) developed an in silico protocol for designing a vaccine against scabies, in which the paramyosin amino acid sequence of *S. scabiei* var. *hominis* was used to design a multi-epitope peptide vaccine with different lengths of epitopes for potential B- and T-cell receptor activation. The vaccine also included a β-defensin-3 adjuvant. However, additional in vitro and in vivo studies are needed to validate and further explore this computational vaccine model for scabies.

Notably, Shen et al. (2023) used a multi-epitope vaccine comprising a recombinant fusion protein containing epitopes of the three proteins, Ss-serpin, Ss-CLP5, and Ss-CLP12, on mangy rabbits. Unfortunately, the reported protective immune response was much lower than that noticed in rabbits immunized with a cocktail of three recombinant vaccine candidate molecules, rSsCLP5, rSsCLP12, and rSs-serpin. The authors emphasized the importance of selecting the right adjuvant for a multi-epitope vaccine to effectively trigger a protective immune response against *S. scabiei*. They also highlighted the significance of screening vaccine candidates, optimizing the vaccine, and determining the appropriate immune dose of the fusion protein to enhance the effectiveness of scabies vaccines.

## Conclusions

While existing scabicide treatments offer relief, the limitations of these methods highlight the urgent need for a scabies vaccine. The ongoing research into scabies vaccines paints a promising picture for the future. Scientists are actively exploring potential antigens and delivery methods and overcoming challenges related to large-scale mite cultivation and animal models. Animal studies are already underway, signifying a crucial step towards a readily available vaccine. Global collaboration between researchers, public health agencies, and funding bodies is essential to ensure successful vaccine development, implementation, and equitable access in all regions. With continued research and international cooperation, scabies vaccines have the potential to revolutionize scabies control strategies, leading to a future where this itchy enemy becomes a relic of the past.

## Data Availability

No datasets were generated or analyzed during the current study.

## Abbreviations

CLPs: Chitinase-like proteins
CS: Crusted scabies
IFN-γ: Interferon-gamma
IL: Interleukin
IVR: Ivermectin
OS: Ordinary scabies
rSs-CLP5: Recombinant *S. scabiei* chitinase-like protein 5
S.: *scabiei Sarcoptes scabiei*
TNF-α: Tumor necrosis factor-alpha
Treg: T-regulatory lymphocytes
